# Shearwaters know the direction and distance home but fail to encode intervening obstacles after free-ranging foraging trips

**DOI:** 10.1073/pnas.1903829116

**Published:** 2019-10-07

**Authors:** Oliver Padget, Geoff Stanley, Jay K. Willis, Annette L. Fayet, Sarah Bond, Louise Maurice, Akiko Shoji, Ben Dean, Holly Kirk, Ignacio Juarez-Martinez, Robin Freeman, Mark Bolton, Tim Guilford

**Affiliations:** ^a^Department of Zoology, Oxford University, OX1 3SZ Oxford, United Kingdom;; ^b^Department of Physics, Oxford University, OX1 3PJ Oxford, United Kingdom;; ^c^British Geological Survey, OX10 8ED Wallingford, United Kingdom;; ^d^Graduate School of Life and Environmental Sciences, University of Tsukuba, Ibaraki 305-8572, Japan;; ^e^Zoological Society of London, Institute of Zoology, NW1 4RY London, United Kingdom;; ^f^Centre for Conservation Science, Royal Society for the Protection of Birds, SG19 2DL Sandy, United Kingdom

**Keywords:** animal navigation, animal cognition, spatial cognition, map and compass, gradient map

## Abstract

While displacement experiments have been powerful for determining the sensory basis of homing navigation in birds, they have left unresolved important cognitive aspects of navigation such as what birds know about their location relative to home and the anticipated route. Here, we analyze the free-ranging Global Positioning System (GPS) tracks of a large sample (*n* = 707) of Manx shearwater, *Puffinus puffinus*, foraging trips to investigate, from a cognitive perspective, what a wild, pelagic seabird knows as it begins to home naturally. By exploiting a kind of natural experimental contrast (journeys with or without intervening obstacles) we first show that, at the start of homing, sometimes hundreds of kilometers from the colony, shearwaters are well oriented in the homeward direction, but often fail to encode intervening barriers over which they will not fly (islands or peninsulas), constrained to flying farther as a result. Second, shearwaters time their homing journeys, leaving earlier in the day when they have farther to go, and this ability to judge distance home also apparently ignores intervening obstacles. Thus, at the start of homing, shearwaters appear to be making navigational decisions using both geographic direction and distance to the goal. Since we find no decrease in orientation accuracy with trip length, duration, or tortuosity, path integration mechanisms cannot account for these findings. Instead, our results imply that a navigational mechanism used to direct natural large-scale movements in wild pelagic seabirds has map-like properties and is probably based on large-scale gradients.

## Significance

Procellariiform seabirds homing from distant foraging locations present a natural situation in which the homing route can become obstructed by islands or peninsulas because birds will not travel long distances over land. By measuring initial orientation from Global Positioning System (GPS) tracks during homing, we found that the Manx shearwater fails to encode such obstacles while homing, implying a navigation system that encodes the direction of home rather than a learned route. Nonetheless, shearwaters timed their journeys home, implying that their navigational system provides them with information about both direction and distance home, providing evidence that for routine, yet long-distance navigation, seabirds probably ascertain homeward direction by comparing their current position and the location of home with 2 or more intersecting field gradients.

Translocation experiments have been crucial in determining the environmental cues that wild animals use to navigate (1–5). How spatial information is represented cognitively in the brains of long-distance navigators, however, remains poorly understood. In contrast to maze-type experiments testing local orientation [e.g., in laboratory mammals (6, 7)] where candidate cognitive mechanisms can be investigated through specially designed behavioral tasks, a challenge to our understanding of long-distance navigation is that displacement experiments have, in general, attempted to investigate only the sensory basis for determining the homeward direction, leaving cognitive factors such as whether an animal encodes distance and an animal’s anticipation of navigational routes largely unaddressed. Hallmarks of spatial cognition might be present, however, in the close observation of free-ranging animal movement, where deviations from maximally efficient trajectories could indicate underlying cognitive and physical constraints (8, 9).

**Fig. 1.**
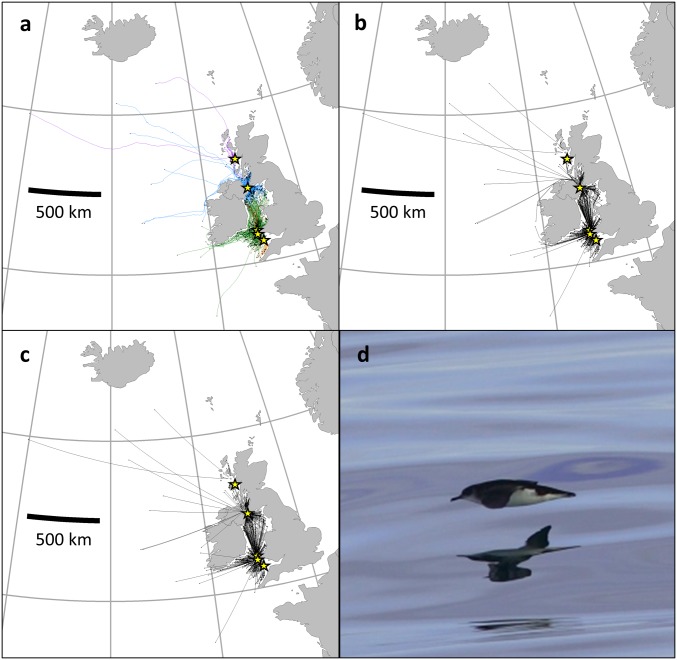
Map of the British Manx shearwater range. (*A*) Shows the GPS tracks of shearwater foraging trips after the algorithmically identified start of homing behavior. Track colors represent different colonies of origin, themselves marked by a yellow star (in descending colony latitude: purple, Rum; blue, Copeland; green, Skomer; red, Skokholm; orange, Lundy). (*B*) The algorithmically calculated shortest route home from the start points avoiding flight over land. (*C*) The beeline home from the start points, not avoiding flight over land. Start points in *A*–*C* are marked by black dots. Map is an azimuthal projection of the northeast Atlantic created using the “maps” package in R ver. 1.1.463. (*D*) An adult Manx shearwater in flight.

The Manx shearwater, *Puffinus puffinus*, a small (400 g) Procellariiform seabird, epitomizes the ability that wide-ranging animals have to orient efficiently on a large scale during exploitation of unpredictable and patchy resources, routinely traveling many hundreds of kilometers to forage during incubation and chick-rearing (10, 11). We analyzed a large extant Manx shearwater Global Positioning System (GPS) tracking dataset (707 foraging trips from 359 individuals) to determine what, at the end of their trips, shearwaters knew about the direction and distance home. To allow cognitive inferences to be made from their homing behavior, we analyzed shearwater homing over a natural contrast with different optimal solutions depending on their cognitive representation of space. Because Manx shearwaters do not fly over land when foraging (12), we focused on those birds beginning their homing journeys from areas where the most direct homeward route was interrupted by an island or peninsula. With complete knowledge of the environment, we would expect shearwaters to home via the shortest distance flying only over water, which, from beyond an island or peninsula, differs from the most geographically direct route between the bird’s location and the colony (Fig. 1). However, if constrained to representing only aspects of the bird’s current location and home rather than a detailed, memorized map, we might expect birds to attempt instead a direct beeline home, failing to anticipate intervening obstacles which they will then be forced to circumnavigate. Making no assumptions about cue use or the cognitive capacity of birds, we consider the Great Circle arc (an orthodrome) between the bird and home as an appropriate beeline. While following a constant bearing route (loxodrome) is another possibility which might be cognitively less demanding (9, 13), this choice is of secondary importance since, over the spatial scale dealt with here, the route differences are negligible (*SI Appendix*) and both the orthodrome and loxodrome are blind to intervening obstacles. Finally, we are able to infer whether birds encode distance home. Because free-ranging Manx shearwaters arrive at the colony shortly after dusk to avoid predation by gulls (Laridae), we measured whether, by estimating the distance over which they must fly, shearwaters were able to judge their departure to achieve timely arrival at the colony (12).

## Results and Discussion

**Fig. 2.**
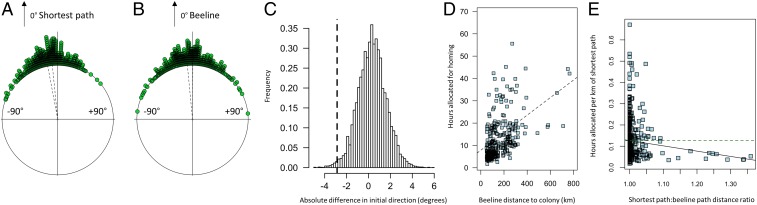
Details of the homing orientation and timing are shown. The initial orientation of shearwaters are shown *A* with respect to the minimum path flying only over water (mean respective orientation: −8.31°; 99% CI: −5.7° to −10.7°) and in *B* to the beeline path blind to intervening obstacles (mean respective orientation: −2.56°; 99% CI: +0.3° to −5.4°). A significant difference between these orientation distributions shows that the shearwaters’ initial orientations were not random with respect to the difference between the beeline and minimum path routes home. The dashed lines show the 95% confidence intervals of each set of relative orientations, respectively. (*C*) To ensure that the beeline orientation observed in *A* and *B* was not the result of a bias in both the initial orientation of shearwaters and a global bias in the difference between the 2 tested routes, we computed a randomization to provide a *P* value. We subtracted each bird’s deflection from the shortest path over only water from its deflection from the beeline, giving negative values for initial orientation closer to the beeline and positive closer to the shortest path over only water. To compute a null expectation for this measure (the histogram shown), we computed this for 10,000 randomly selected startpoint minimum paths and beeline routes. The black line shows the observed “beeline closeness” for startpoints where the resulting difference in route length was >5%. (*D*) The hours that shearwaters began their journey before midnight as a function of the beeline distance to the colony. (*E*) Scatter plot showing the decrease in time allocated to homing per unit distance as the ratio between the shortest path and beeline routes becomes large.

We first identified the start of homing algorithmically for each free-ranging shearwater GPS track. The start of homing is signified by a distinct change in behavior (*Materials and Methods*) as birds go from spending most of their time foraging or resting to the majority of time in fast, directed flight. Birds for whom the shortest potential route home was an uninterrupted traverse of open ocean (*n* = 370), the initial homing orientation had a mean bearing of −0.80° from the beeline between themselves and home, with an average deflection of 15.7°, showing that shearwaters start homing from afar with an accurate estimate of the homeward direction (Fig. 1*A*, Movie S1). For homing tracks starting beyond islands or peninsulas (*n* = 337), however, the beeline is interrupted by terrain over which shearwaters will not fly, so we also calculated algorithmically a shortest feasible route home without crossing land (Fig. 1*B*). For these birds where the beeline and the minimum path differed, mean deflections were, on average, significantly closer to the beeline (2.60° to the beeline, Fig. 2*A*) than the minimum path [8.21° deviation, Fig. 2*B*, Watson–Williams test, *F*(1, 672) = 13.39, *P* < 0.001], implying that orientation decisions were often blind to flight-path obstacles. Since many of the interrupted routes involved only a small detour, we computed this analysis for the subset of trips where the path lengths of the 2 routes differed by more than 1% [*n* = 191, initial orientation: 1.47° from beeline, 7.94° from minimum path; Watson–Williams test, *F*(1, 380) = 18.65, *P* < 0.0001] and 5% [*n* = 79, initial orientation: 1.03° from beeline, 9.83° from minimum path; Watson–Williams test, *F*(1, 156) = 8.76, *P* < 0.01]. By randomizing the identity of each track’s beeline and minimum path, we were able to show that orientation apparently blind to intervening obstacles was not an artifact of an overall bias in orientation at the start of homing coupled with an overall directional bias between the required orientation for the beeline and minimum path home (where the path lengths of the 2 routes differed by greater than 1% (*n* = 191, 10,000 repetitions, 2-tailed *P* < 0.05) and 5% (*n* = 79, 10,000 repetitions, 2-tailed *P* < 0.005, Fig. 2*C*). Thus, on average, shearwaters computed accurately the direction home, but did not anticipate the intervening terrain.

To assess whether shearwaters also knew their distance from the colony at the start of homing, we next analyzed whether they began homing earlier in the day when they had farther to go. For birds homing along uninterrupted routes (*n* = 369), we found a strong relationship between time allocated to homing and starting distance from the home colony (analyzed with a linear mixed model, for which the parameter beeline distance, χ^2^ = 113.5, *P* < 0.0001; Fig. 2*D*). Similarly, for birds homing from beyond topographic obstacles (*n* = 338), we found that both the beeline distance and the minimum path over water predicted the time that homing began, since they are correlated. While the quantitative differences between these 2 kinds of paths are relatively small, they do imply cognitively different states: the minimum path implies knowledge of the actual flight distance and therefore memory of that path from previous experience; the beeline implies knowledge of absolute geographical distance, whether or not it is actually flyable. So we compared the 2 kinds of path using nonnested models containing either the beeline or the minimum path as a predictor and time allocated to homing as the response. We found that, for all trajectories, and those where the predicted difference between the paths was greater than 1%, the beeline was the best predictor [all trajectories: *n* = 338, Δ Akaike information criterion (ΔAIC) = −3.37; 1% difference: *n* = 191, ΔAIC = −3.30]. The difference in predictive power between our 2 models increased even further when we analyzed only those trajectories with a greater than 5% difference in predicted path length (*n* = 79, ΔAIC = −8.91). Fig. 2*E* shows graphically how shearwaters progressively under-allocate time to the homing journey as the ratio between the beeline and the minimum path increases [a relationship which is significant when tested using a likelihood ratio test between nested models with and without path ratio as a predictor, χ^2^(1) = 5.9, *P* < 0.015]. We did not find that the head–tail component of the wind vector experienced by shearwaters at the start of homing explained significantly more variation in the timing of homing than our model including only beeline distance (*n* = 707), χ^2^(1) = 2.23, *P* > 0.1. While a detailed analysis of how wind influences homing decisions is beyond the scope of this study, this finding, in combination with our finding that birds oriented along the beeline, implies that neither Manx shearwaters’ homing trajectory nor their precise timing of homing was primarily dictated by the wind. This is in contrast to some other Procellariiform seabirds (14), but is consistent with the finding that Manx shearwaters’ migratory routes are less influenced by wind than those of other shearwater species, most probably owing to higher wing loading and higher proportion of flapping flight (15). Therefore, independently of wind and other factors that might influence the timing of homing, shearwaters appear to use knowledge of the distance to time their journeys home, and there is evidence to suggest that this distance estimation, like orientation, is probably blind to intervening obstacles. Thus shearwaters underestimate the time actually needed to home when a land obstacle will be encountered on the way. Together, the orientation and timing of shearwater homing trips is therefore consistent with birds using knowledge of the beeline between themselves and the colony, blind to intervening obstacles, to judge their orientation toward home and that this is the most likely trajectory involved in their distance estimation also.

From a cognitive point of view, our study shows principally that shearwaters execute accurate long-distance homeward orientation involving both distance and direction estimation, but remain apparently blind to the presence of intervening obstacles requiring detours. This, in principle, could be achieved in several cognitively different ways, and we explore three possibilities. First, perhaps least likely, is the possibility that they have formed through previous experience a large-scale familiar area map encoding the unique features of remote locations from which they have homed before and then encoded these in a sparse representation retaining only vectoral information. However, this would essentially require memorizing information pertaining to routes that had not actually been experienced, so it is unclear what learning mechanism could achieve this. The second possibility is that the birds, like ants (16, 17), use path integration to compute a running egocentric homing vector by registering the rotations and translations that they experience on their outward journey, which would naturally be blind to obstacles in the homing path. Using compass-based information as a heading indicator (18) to measure rotations, and mechanisms such as optic flow across the ocean surface, might allow path integration to obviate the large cumulative error associated with using vestibular information. Nevertheless, all path integration suffers from error accumulation as a result of noise in the perception, processing, storage, and outputting of angular and distance estimates (19), so the long and tortuous movements involved in oceanic foraging journeys, which probably include periods of sleep and take place within and above 2 separate moving fluid media, would not seem to favor accurate path integration. Since shearwater foraging trips vary in total length traveled, duration, sinuosity, and beeline homing distance, we were able to examine directly, for trips with uninterrupted trajectories (*n* = 369), whether homing orientation accuracy deteriorated as these 4 variables increased. Despite the large sample size, we found no relationship between homing accuracy and sinuosity, time or the total distance traveled before homing: path length, *F*(10, 359) = 1.96, *P* = 0.051; total sinuosity, *F*(10, 359) = 1.49, *P* = 0.138; time spent before homing, *F*(10, 359) = −0.467, *P* = 0.64 (*SI Appendix*, Fig. S2). The very small and nonsignificant relationship with distance traveled would require an error accumulation rate of less than 0.006° per kilometer (the modeled effect size), implying that homing orientation error cannot be explained by the operation of path integration alone. In contrast, we did find a significant but negative relationship between beeline homing distance and orientation error, *F*(10, 359) = −3.94, *P* < 0.0001, which is inconsistent with path integration but consistent with predictions of some true navigation models (20). In some versions of the olfactory map, for example, the spatial resolution of location finding is thought to allow increasingly poor homeward orientation from places close to home (20). These findings support a third possibility: that shearwaters might be using true navigation to calculate their location relative to home by comparing the current values of (at least) 2 intersecting environmental gradient fields, the characteristics of which they had learned in their familiar area closer to the colony (8). This cognitive possibility has been most intensively investigated in homing pigeons (21, 22), where it is usually assumed to account for the ability to orient homeward following artificial displacement to unfamiliar locations (22–24). However, because the displacement paradigm cannot readily assess a true navigator’s distance estimation, only its orientation, the simpler navigational mechanism of only encoding direction to home from the pattern of environmental gradients has never been empirically ruled out, and indeed some theoretical models of pigeon navigation explicitly exclude the encoding of distance (20, 25). In addition, because experimentally displaced animals are effectively forced to make orientation decisions without any outward journey information, it remains possible that apparent homeward orientation from unfamiliar sites can arise as an artifact of experimental displacement through generalization of familiar area homing mechanisms and is not actually a functional navigational ability (26). While it remains possible that shearwaters could use more than one of these mechanisms, our findings are probably the clearest evidence to date that a wild bird uses gradient-based navigation to home and that this has map-like properties. Both future displacement experiments and careful analysis of natural foraging trips, combined with sensory manipulations and natural contrasts, might provide crucial insight not only about the cognitive representation of space, but also about specific cue-use underpinning map and compass navigation in highly mobile oceanic navigators (9, 13, 27).

## Materials and Methods

### Data Availability.

Data have been deposited in Movebank (https://doi.org/10.5441/001/1.k20j58qt) and are available for download (28).

### Ethics Statement.

GPS tracking of Manx shearwaters was approved by Oxford University’s Animal Welfare and Ethical Review Board.

### Manx Shearwater Tracking.

Between 2008 and 2016, Manx shearwaters were tracked on Skomer Island (51°44′15.7″N, 5°16′58.8″W) and Skokholm Island (51°41′38.6″N, 5°17′7.5″W), South Irish Sea, Wales; Lighthouse Island (51° 41′ 38.5′′N, 5°31′32.9′′W), Copeland Islands, Northern Ireland; Rum (56°58′59.4′′N, 6°17′41.3′′W), Hebrides, Scotland; Lundy (51°10′7.6′′N, 4°40′7.8′′ W), Bristol Channel, England. These 5 islands widely sample the Irish Sea distribution of the species and across the species’ breeding season including tracks from incubation and chick-rearing stages. We used I-gotU gt-120 GPS devices scheduled to take fixes at 5-, 10-, or 15-min intervals. Devices were attached dorsally to the birds using TESA marine tape as in Guilford et al. (29). GPS devices comprised no more than 4% of each bird’s body mass.

### Track Selection.

Only tracks which went out of sight of the home colony (∼40 km) were included since we were interested in inferring aspects of a shearwater’s map rather than its ability to judge distance to home from closely associated topographic features. This gave a total of 731 tracks for analysis.

### Track Processing and Identifying the Homing Point.

Tracks were first interpolated to exactly 5-min intervals using cubic spline interpolation (30). To identify the point at which the birds began to home, we implemented a Douglas–Peucker line simplification algorithm (31) to identify break points in the curve given by the relationship between distance from the home colony over time. We assume that the decision to begin homing occurs somewhere between the point where the bird is farthest away from the colony and when it arrives close to the home colony (within 40 km) and so sought to find break points in this section of track for each trip. The Douglas–Peucker algorithm is used here, and in other fields, to reduce the complexity of shapes (e.g., in cartography) and finds a curve similar to the input curve but with fewer points—those which are of most importance for defining its shape (32). The result is that the homing trajectory is broken into temporal units where progression toward home over time is consistent, each unit defined by a break point at its start and end. The number of break points obtained varied depending on the sensitivity parameter of the algorithm, and so the Douglas–Peucker algorithm’s sensitivity parameter was increased systematically from that which gave no break points until the gradient between last break point and the end of the track was greater in steepness than that of the farthest distance and the end of the track. The break point that gave the steepest final section was then taken as the start of homing. This therefore makes the assumption that the decision to home happens once and that it occurs between the point where the shearwater is farthest away and when it arrives back at the colony, but provides an objective and repeatable position for where homing begins.

### Validating the Point of Homing.

If the break point identified as homing identifies a real biological phenomenon, then we would expect shearwater behavior either side of this break point to change considerably and more so around the identified breakpoint than randomly selected points across the same constricted section of the trajectory. Therefore, to validate the point identified as the start of homing, we identified at-sea behavior of the shearwaters by classifying them into discrete behavioral states using a Hidden Markov Model (HMM) fitted to speed and turning angle of the trajectories (8, 16–19). We computed an HMM for different numbers of states from 1:10 and then chose the number of states which were the best compromise between model fit and explanatory power by identifying an elbow in the increasing log-likelihood of models (10). As in previous studies on Manx shearwater (10, 33), 3 states were best supported by our data. Our high-speed cluster was interpreted as directed flight, midspeed as foraging, and low speed as resting on the water. We then tested whether the proportion of directed flight, foraging, and resting changed consistently with homing (more flight, less foraging and resting) from the period before and the period after homing (the first 25% of the homing track and the same time before the start of homing) and compared this to randomly selected 25% sections of track (1,000 times).

### Beeline and Minimum Path Distance.

The beeline route was defined as the Great Circle arc between each bird’s location and its colony at the point that homing began and was calculated with the Vincenty ellipsoid Great Circle formula implemented in the R package “geosphere.” The minimum path route was the route from the same point that a bird would need to travel to get home flying only over water, navigating around land features. This was done initially by using the “raster” and “gdistance” packages in R to find the minimum cost route over a rasterized map of the northeast Atlantic from the start point to the colony with land set as a high cost to traverse. We applied an inbuilt (16-neighbor) correction for Manhattan distance to Euclidean distance (a problem inherent with rasterized distance measurements), but this needed to be refined further and so we then applied an algorithm that iteratively placed Great Circle arc routes along the resulting path to shorten the path until part of the path crossed land. All measurements were made using the Vincenty ellipsoid Great Circle implementation in the R package geosphere.

### Wind Data.

We used the L3.0 Cross-Calibrated Multi-Platform Ocean Surface Wind Vector V2.0 wind data from the National Aeronautics and Space Administration (accessed August 15, 2017; ref. 34), giving a wind vector every 6 h at a grid resolution of 0.25°. We bilinearly interpolated wind direction and magnitude in space and then interpolated the resultants in time to give a wind direction and magnitude for the time and position that each of the homing trips in the study began. We computed the component of the wind vector along the headwind–tailwind axis to address whether this influenced the time when shearwaters began to home.

### Measuring Initial Orientation.

For homing positions that started close to a coast, the coast often constrained the possibility for birds to follow the beeline (i.e., there was only a short distance for which the beeline route was possible), and so we analyzed the homing track orientation from the start of homing to the halfway point between the birds’ position and the position at which they would reach land were they to follow the beeline route (i.e., the point at which the beeline route becomes impossible).

### Statistics: Orientation.

To analyze whether birds were oriented initially toward the minimum path or the beeline route home, we first normalized the birds’ initial homing orientation with respect to our 2 types of homeward path so that the birds’ initial orientations were positive and negative deflections were around 0, first set as the beeline and then as the minimum path home. We compared these 2 distributions of orientations by bootstrapping 95 and 99% confidence intervals of the mean and then testing for a difference between them using a circular analysis of variance (Watson–Williams test). Since, for most starting positions, the orientations of the beeline and minimum path route were relatively close, we then subtracted each bird’s minimum path deflection from its beeline deflection, giving negative values for birds that were closer to the beeline and positive values for birds that were closer to the minimum path. To calculate a null expectation and *P* value, we randomly sampled the deflections from the beeline and minimum path (essentially randomizing pairs) to give an expected distribution for orientations if they did not lie closer to the beeline or minimum path and calculated the proportion of times that the randomized median was equal to, or lower than, the observed median. We repeated this analysis for all tracks for which there was a predictive difference between the 2 routes and then for those tracks where the predictive difference in distance was greater than 1 and 5% of the beeline distance. Medians were chosen since some of the birds were poorly oriented with respect to both distances, and the magnitude of deflection of those birds would weight more highly the result than others. A median allowed us to find the most common strategy across the birds in our dataset.

### Statistics: Timing of Homing.

We used Linear Mixed Models (LMMs) to analyze whether the time that shearwaters began to home was best predicted by the beeline distance or the minimum distance that shearwaters would need to travel to arrive home before sunset. We fitted an LMM with the hours before midnight that shearwaters began to home as the response variable. The model included island, breeding stage [incubation, chick-rearing, or chick-rearing with no chick (failed breeder)] as fixed effects and bird identification as a random, intercept-only effect. To assess which model best predicted the time that shearwaters began to home and thus the distance estimate made by shearwaters, we compared these 2 nonnested models using ΔAIC.

## Supplementary Material

Supplementary File

Supplementary File
