# Supplementary material for: Shearwaters know the direction and distance home but fail to encode intervening obstacles after free-ranging foraging trips
Source: Proc Natl Acad Sci U S A. 2019 Oct 7;116(43):21629–33. doi: 10.1073/pnas.1903829116 (PMC6815147; doi:10.1073/pnas.1903829116)
Supplement: Supplementary File [file pnas.1903829116.sapp.pdf]

PNAS
www.pnas.org

Supplementary Information for

Shearwaters know the direction and distance home, but fail to encode intervening obstacles after free-ranging foraging trips

*O. Padget[1], G. Stanley[2], J.K. Willis[1], A. Fayet[1], S. Bond[1], L. Maurice[3], A. Shoji[1], B. Dean[1], H. Kirk[1], I. Juarez-Martinez[1], R. Freeman[4], Bolton, M.[5] and *T. Guilford[1]

[1]Department of Zoology, Research and Administration Building, 11a Mansfield Road, Oxford Ox1 3SZ.
[2]Department of Physics, Parks Road, Oxford, Ox1 3PJ.
[3]British Geological Survey, Benson Lane, Wallingford Ox10 8ED.
[4]Institute of Zoology, Zoolical Society of London, Regent's Park, NW1 4RY.
[5]RSPB Centre for Conservation Science, The Lodge, Potton Road, Sandy, Bedfordshire, SG19 2DL.

***Corresponding Authors:** 1. Oliver Padget, oliver.padget@zoo.ox.ac.uk, Department of Zoology, Research and Administration Building, 11a Mansfield Road, Oxford, Ox1 3SZ. +44 (0) 7745 211223. 2. Tim Guilford, tim.guilford@zoo.ox.ac.uk, Department of Zoology, Research and Administration Building, 11a Mansfield Road, Oxford, Ox1 3SZ.

This PDF file includes:

Supplementary text
Figure S1
Figure S2
Captions for movie S2

Other supplementary materials for this manuscript include the following:

Movie S1

www.pnas.org/cgi/doi/10.1073/pnas.1903829116

Comparison of orthodrome and loxodrome routes home

The shortest route home following a constant bearing (the loxodromic route) was calculated using the R package ("geosphere"). This was then used for the following analyses:

The mean angular difference between orthodromic and loxodromic routes home was 0.36°. Correspondingly, there was almost no difference in mean deflection from Great Circle arc (n = 707, -0.80°) and from the loxodrome (n = 707, -0.87°). Histogram of deflections is shown in Figure S1.

The loxodrome did not predict timing of homing significantly better than the Great Circle arc (GCA), either for all tracks (n = 707, ΔAIC = 0.34) or for those beginning their homing journeys from beyond intervening obstacles (n = 337, ΔAIC = 0.16).

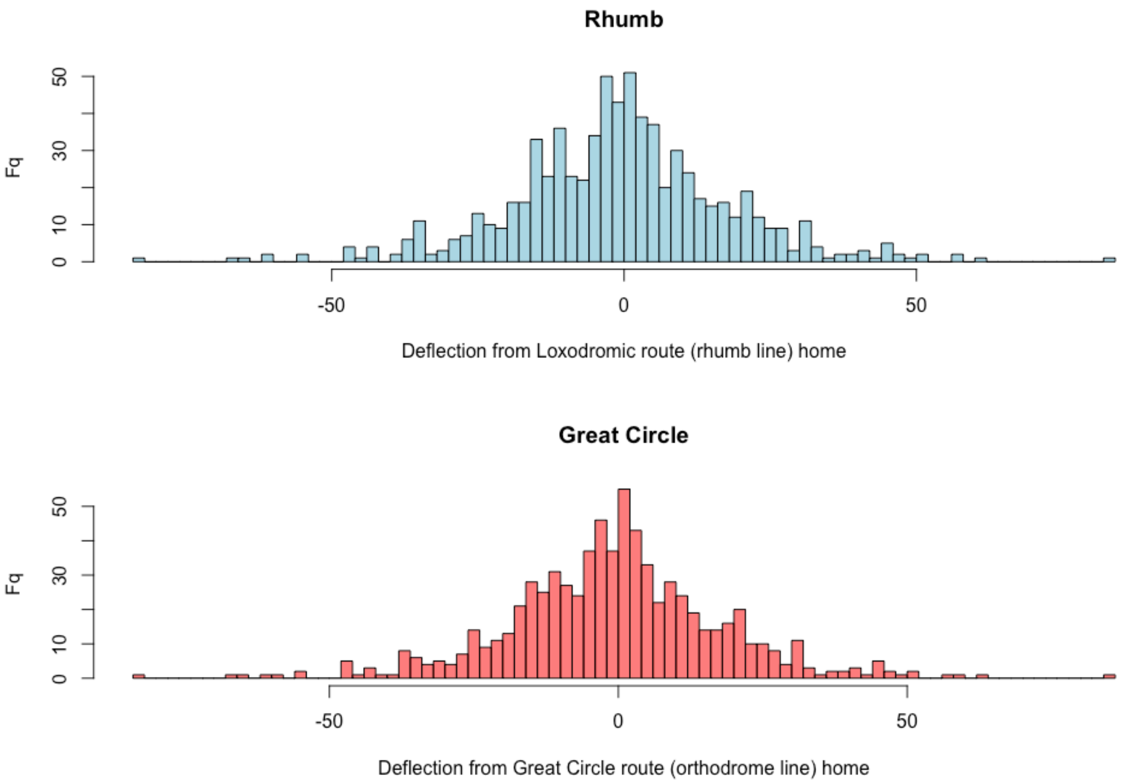

Figure S1. Histograms showing deflections, in degrees, from the loxodromic route home (upper panel) and the Great Circle arc, or orthodrome, route home (lower panel).

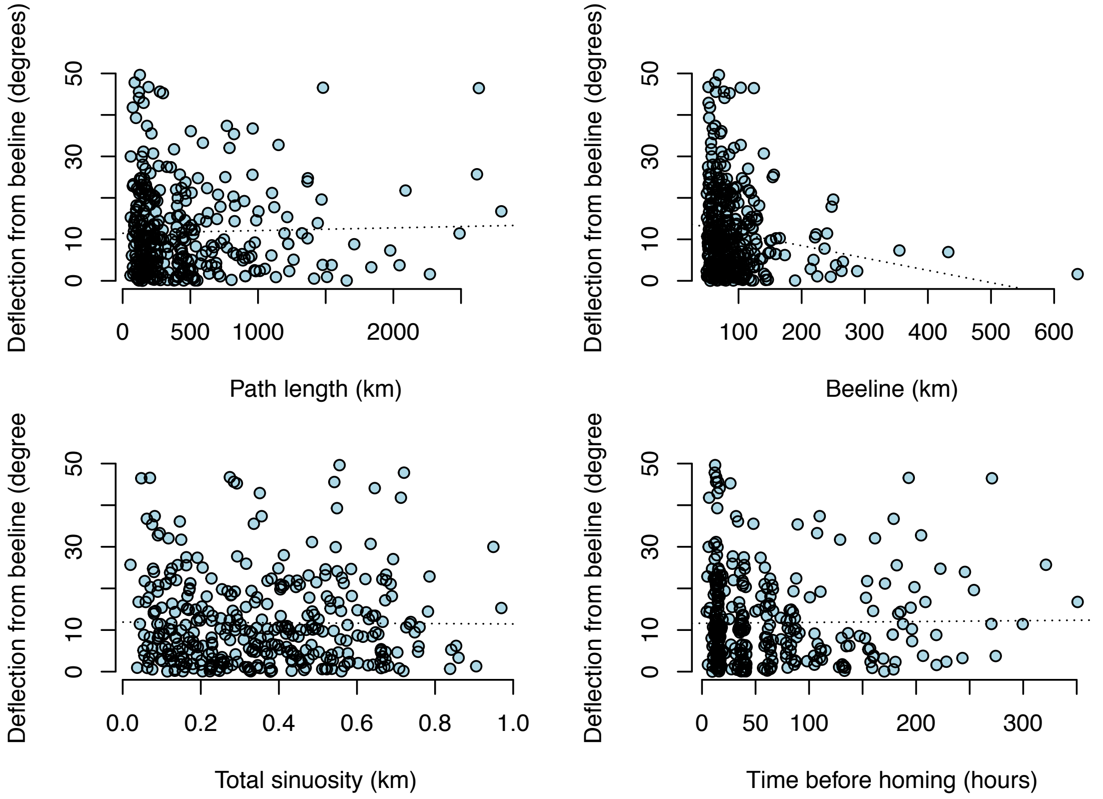

Figure S2. Scatter plots showing the deflection from the beeline in initial orientation as a function of: (i) total distance travelled (path length); (ii) beeline distance from target; (iii) total sinuosity (path length/beeline); and (iv) time spent on trip up to homing. Lines shown are linear regressions for each comparison. Effects of these four variables on homeward deflection were analysed using a linear model with deflection as a response and all four variables as predictors. The results of the linear model show no effect on initial deflection of path length ($F_{10,359} = 1.96$, $p = 0.051$), total sinuosity ($F_{10,359} = 1.49$, $p = 0.138$) or time before homing ($F_{10,359} = -0.467$, $p = 0.64$). There was a significant effect of beeline distance on homing accuracy ($F_{10,359} = -3.94$, $p < 0.0001$) with birds being better oriented on average by 0.05 degrees per kilometre distance between the colony and the start of homing.

Legend for Supp. Mat. Movie.

Movie clip shows a shearwater first heading out to the Atlantic foraging from Copeland Island, Northern Ireland. The bird then forages, exhibiting tortuous and slow movement before a distinct change in behaviour as the bird begins to home back to its colony, along the beeline, failing to anticipate the Island of Ireland.
